# The Annual Report for 1885 of the Surgeon-General of the Army

**Published:** 1885-12

**Authors:** 


The Annual Report of the Surgeon-General of the United States Army, 1885, is a pamphlet of 93 pages, which presents the medical statistics of the army and details as to the work of the Surgeon-general's office, with the precise carefulness which has marked all the publications having the same imprint for all the years since the civil war. The mean strength of the army for the year is set down as 24,035. This body of men furnishes 36,829 records of admission for hospital or other treatment, with 263 deaths. The order of the diseases, as they occurred, possesses some interest, as the soldiers were presumably scattered throughout all parts of the American Union, and hence the average, in some small measure, represents the average of diseases of men in similar hygienic environment all over the country, women, of course, being excluded. First come traumatisms, which may be, perhaps, set down as in a sort peculiar to the army life; second, diseases of the respiratory tract; next, those diseases of the gastro-intestinal tract producing diarrhœa and kindred maladies; next, the malarial group of troubles; and then, in the order named, digestive disorders exclusive of tonsilitis; nervous affections, from headache to those of a severer grade, exclusive of insanity; disorders connected with alcoholism; the venereal diseases, exclusive of constitutional syphilis; tonsilitis; and lastly, cutaneous disorders. The army medical museum received a large addition during the year. It is interesting to note that the 3rd medical volume of the Medical and Surgical History of the War, the last of this series, will probably be completed during the coming winter.

